# Lifestyle Clusters and Cardiometabolic Risks in Adolescents: A Chinese School-Based Study Using a Latent Class Analysis Approach

**DOI:** 10.3389/fped.2021.728841

**Published:** 2021-12-16

**Authors:** Weiying Zhao, Danyan Su, Luxia Mo, Cheng Chen, Bingbing Ye, Suyuan Qin, Jie Liu, Yusheng Pang

**Affiliations:** Department of Pediatrics, The First Affiliated Hospital of Guangxi Medical University, Nanning, China

**Keywords:** lifestyle behaviors, cardiovascular risk, latent class analysis, adolescent, lifestyle clusters

## Abstract

**Background:** Unhealthy dietary and lifestyle behaviors are associated with a higher prevalence of non-communicable chronic diseases and higher mortality in adults. However, there remains some uncertainty about the magnitude of the associations between lifestyle behaviors and cardiovascular factors in adolescents.

**Methods:** We conducted a school-based cross-sectional study of 895 Chinese adolescents aged 15–19 years. They participated in a questionnaire survey, physical examination, and blood sample collection. Latent class analysis (LCA) was used to identify heterogeneous subgroups of lifestyle behaviors. A set of 12 latent class indicators, which reflected lifestyle behaviors including dietary habits, physical activity, sleep duration, screen time, and pressure perception, were included in the analysis. Logistic regression analysis was performed to determine whether the derived classes were related to a cardiometabolic risk.

**Results:** In total, 13.7 and 5.6% of the participants were overweight and obese, respectively, and 8.4 and 14.1% reported having pre-hypertension and hypertension, respectively. A two-class model provided the best fit with a healthy lifestyle pattern (65.8%) and a sub-healthy lifestyle pattern (34.2%). There were more female participants with a healthy lifestyle (56.2 vs. 43.8%), whereas there were more males with a sub-healthy lifestyle (45.4 vs. 54.6%), (all *P* = 0.002). Increased risk of cardiometabolic abnormality (BMI categories, blood pressure and lipids) was not significant across lifestyle patterns, except for waist circumference (70.5 vs 69.1 cm, *P* = 0.044). There was no significant difference in physical activity and intake of fruit and vegetable between the two patterns.

**Conclusion:** Primary prevention based on lifestyle modification should target patterns of behaviors at high risk in adolescents. Due to the complex effect of lifestyle clusters on cardiometabolic risks, well-designed and prospective studies in adolescents are needed in the future.

## Introduction

Childhood and adolescent obesity are known to be associated with a range of cardiovascular complications, and regarded as a global public health concern (1). Obesity in children and adults is identified as a primary risk factor for hypertension (2). A recent systematic review and meta-analysis reported that elevated blood pressure (EBP) in childhood or adolescence is strongly associated with cardiovascular morbidity and mortality in adulthood (3). Atherosclerosis begins in childhood, and cardiovascular trajectories from early life onward may be associated with a risk of cardiovascular disease (CVD) in later life (4, 5).

The Global Burden of Disease Study has presented data supporting the fact that the prevalence of childhood obesity has more than doubled since 1980, with the overall prevalence of overweight and obesity being as high as 23% in 2015 (6). The incidence of metabolic syndrome in children and adolescents with obesity ranges from 6 to 39%, depending on the definition applied (7, 8). When defined according to the American Academy of Pediatrics (AAP) guidelines, EBP is prevalent in 8.6% of the pediatric population in 2017 (9). In China, the prevalence of pediatrics EBP is 9.8% (10), indicating the importance and urgency of addressing cardiometabolic abnormalities in adolescents. Unfortunately, the mechanism regulating cardiovascular risks during this period is yet to be fully elucidated. Unhealthy dietary and lifestyle behaviors are known to be associated with a higher prevalence of non-communicable chronic diseases (NCCDs) and increased mortality in adults (11). However, the magnitude of the associations between lifestyle behaviors and cardiovascular factors in adolescents remains unclear to date.

Lifestyle behaviors are multidimensional and complex, and consist of different elements including dietary habits, physical activity (PA), sleep duration, and emotional stress. All these factors have been suggested to play a role in the development of obesity and related CVD in children (12). Previous studies have generally focused upon the impact of individual lifestyle behaviors rather than the overall effects of lifestyle factors on obesity and cardiovascular risk. However, any element does not exist in isolation and contributes to the development of CVD, and therefore it is more meaningful to study the effects of a combination of lifestyle factors (13, 14). Nonetheless, studies on the impact of the overall lifestyle behavior on health are scarce, and some studies have only focused on the role of dietary patterns (15–17).

Latent class analysis (LCA) is a statistical technique that identifies a categorical latent class and investigates whether there are subgroups defined by a combination of the observed variables, without mandating consideration of the outcomes (18). It is a data-driven exploratory method used to group similar individuals. It can offer important implications for health promotion strategies by identifying sub-phenotypes of disease. Although this approach has identified lifestyle behavioral clusters based on varying lifestyle characteristics and has demonstrated certain connections between clusters and obesity (19–24), studies exploring connections between clusters and cardiometabolic outcomes are scarce.

Approximately 1.8 billion of the population in 2012 were adolescents (10–24 years). Late adolescence (15–19 years) is a stage characterized by pubertal maturation. Lifestyle habits are established during this period and tend to be carried through into adulthood, even lasting throughout the lifetime (25). Interventions targeted at adolescents allow health systems and policies to have the maximum impact on optimizing health in the preceding years. This study aimed to identify modifiable lifestyle behaviors and explore the best way for optimal intervention in Chinese adolescents. Toward this goal, we used latent class analysis to identify sub-phenotypes of lifestyle behaviors and test their association with obesity, blood pressure, and lipids.

## Methods

### Study Design and Subjects

This cross-sectional study was conducted in September 2020. The subjects were high school freshmen participating in a medical examination for enrollment in a large-scale boarding school in Nanning, Guangxi, Southwest China. The project consisted of three stages, namely, questionnaire survey, physical examination, and blood sample collection. Questionnaires were sent home and filled out by the students with their parents' help, and unclear information was clarified via telephone. Trained medical staff undertook the physical examinations and blood collections. Among the 1,291 participants initially screened, we excluded adolescents with (1) medical history of acute infectious disease; heart, brain, and renal disease; and drug use; (2) unable to participate in PA freely; and (3) incomplete data about lifestyle behaviors. Of 895 remaining subjects, 592 subjects consented to undergo blood tests.

### Lifestyle Assessment

#### Dietary Assessment

A simplified semi-quantitative food frequency questionnaire was used to estimate usual dietary intake over the last 7 days. The FFQ included 20 locally common items that are linked to CVD. These included snail noodles, pickled vegetables, ham sausage, puffed food, dairy products, lean meat, eggs, beans, fish, sweets, desserts, cakes, rice, French fries, hamburgers, potato chips, fried food, fresh vegetables, fresh fruits, and sugar-sweetened beverages (SSB). The intake frequency was investigated by the number of times in a week each food was consumed, with options of “Never,” “1–2 times,” “3–6 times,” and “more times daily.” The food items were classified into five categories based on their content. High-salt foods included snail noodles, pickled vegetables, ham sausage, and puffed food. High-protein foods included dairy products, lean meat, eggs, beans, and fish. High-carbohydrate foods included sweets, desserts, cakes, and rice. High-fat foods included French fries, hamburgers, potato chips, and fried food. Given that the specific serving and portions were not available, the consumption of each food group was assessed using the sum of all items from each group. They were then divided into the low and high exposed groups based on the frequency of consumption.

#### Measurement of Lifestyle Behaviors

PA that lasted at least 30 min in the previous 7 days was recorded. The intensity was classified as (1) moderate (i.e., brisk walking, biking, climbing stairs, and swimming) and (2) vigorous PA (i.e., playing basketball, playing football, running, rope skipping, spinning, and dancing). The estimated PA was calculated by summing the time spent doing moderate and vigorous PA, and the intensity was dichotomized as high (>3 days per week) and low (≤ 3 days per week). Late-night eating (defined as eating before going to bed or nocturnal snacking) was assessed by asking how many days per week the participants consumed snacks at night. This was also categorized into two: high (>3 days per week) and low (≤ 3 days per week). Sleep duration was evaluated according to the usual time for sleeping at night (26). It was divided into three categories:>8 h, 6–8 h, and <6 h, with <6 h considered as a short sleep duration for adolescents, and thus classified as low exposure. Screen time was evaluated according to the total time spent per day using electronic devices, such as TVs, personal computers, mobile phones, and iPads. It was also divided into two categories based on the frequency (2 h per day). Regular dining was defined as having three main meals per day; that is breakfast, lunch, and dinner per day at a relatively fixed time. SSB consumption was assessed by the number of times all SSB (i.e., juice, soda) consumed in a regular week (usual portion size per time = 250 ml). Therefore, the consumption of SSB was categorized as “High” intake (≥3 times/week) and “Low” intake (0–2 times/week). Pressure perception was investigated through a subjective question in the self-designed questionnaire. The participants were asked the following questions: how often do you feel anxious, depressed, or under pressure from your studies or daily life. The answer options included “never,” “rarely,” “occasionally,” “often,” and “always”. Then “often” and “always” were considered as “Yes”, and the other options are considered as “No”.

### Anthropometric Measurements

The anthropometric measurements were performed by trained medical staff according to standard protocols. Weight and height were measured with light clothing and without shoes. Waist circumference (WC) was measured at the midpoint between the right lower rib and the iliac crest, and after a slight breath out. Weight was measured in kilograms (kg) to the nearest 0.01 kg. Height and WC were measured to the nearest 0.1 centimeter (cm). Body mass index (BMI) was calculated as weight in kg/(height in meters)^2^. Overweight and obesity were defined as BMI above the 85th and 95th percentiles of age- and sex-specific reference values of Chinese adolescents (27). Abdominal obesity was defined as a WC above the 85th percentiles of age- and sex-specific reference values (28). Systolic and diastolic blood pressure (SBP and DBP) were measured using a digital automatic monitor (OMRON, HEM-7124) on the right arm after 15 min of seated rest and measured three times over 5 min period. The averaged of the last two BP readings were used in the final analysis. Prehypertension and hypertension were defined as SBP and/or DBP above the 90th and 95th percentiles of age-, sex-, and height-specific reference values of Chinese adolescents, respectively (29).

### Blood Sample Collection

The Lipids in non-fasting blood samples collected by venipuncture were measured. The levels of high-density lipoprotein cholesterol (HDL-C), low-density lipoprotein cholesterol (LDL-C), total cholesterol (TC), triglyceride (TG), and uric acid (UA) were measured by using enzymatic methods with an autoanalyzer (Type 7170A; Hitachi Ltd., Tokyo, Japan) in our Clinical Laboratory Center. The lipid assay kits were provided by KANGBOLAI company, and the uric acid kit was provided by BIOSINO BIO-TECHNOLOGY AND SCIENCE INC company.

### Statistical Analysis

Descriptive statistics were presented as percentages for categorical variables and as means and standard deviations (medians and interquartile ranges) for continuous variables. Between group, comparisons were performed using the non-parametric Mann–Whitney *U* or two-sample *t*-test depending on normality, and chi-squared test for categorical variables, respectively. Twelve lifestyle behaviors were considered as observed variables in the latent class analysis model, and a series of latent class models were fitted. Supplementary Table S1 shows the information of the Mplus code used for the LCA. The latent class model estimation was based on full information maximum likelihood methods. The criteria for model selection were based on Akaike's information criterion (AIC), Bayesian information criteria (BIC), adjusted Bayesian information criteria (aBIC), and entropy. Lower values of AIC, BIC, and aBIC, and a higher entropy indicated a better model fit. The optimal number of classes was also determined using the bootstrap likelihood ratio tests (BLRTs). Once we established the number of classes, and next we tested the associations between class and cardiovascular outcomes (obesity and hypertension) by binary logistic regression models, adjusting for age and sex. All statistical analyses were performed using Mplus 8.3 and SPSS 24.0. All tests were two-sided, and *P* < 0.05 was considered statistically significant.

## Results

### Descriptive Statistics

The difference between the included and excluded adolescents are shown in Supplementary Table S2. There was no significant difference in sex, age, BMI, and BP between the two groups. In total, 895 adolescents aged 15–19 years in the final analysis represented the overall population for the general features. The average age was 16.0 ± 0.5 years. In total, 13.7 and 5.6% of the participants were overweight and obese, respectively, and 8.4 and 14.1% reported having pre-hypertension and hypertension, respectively. The characteristics of the participants are presented in Table 1.

**Table 1:** The characteristics of participants in the study.

**Parameters**	**Category**	***N* = 895**	**Percent (%)**
Ethnic	Han	388	43.4
	Others	507	56.6
Sex	Female/male	470/425	52.5/47.5
Weight status	Obesity	50	5.6
	Overweight	123	13.7
	Normal	722	80.7
Central obesity	Yes	195	21.8
	No	700	78.2
Blood pressure status	Hypertension	126	14.1
	Pre-hypertension	75	8.4
	Normal	694	77.5
High-salt foods	Low	688	76.9
	High	207	23.1
High-protein foods	Low	500	55.9
	High	395	44.1
Fruit and vegetable intake	Low	213	23.8
	High	682	76.2
High-carbohydrate foods	Low	609	68.0
	High	286	32.0
High-fat foods	Low	734	82.0
	High	161	18.0
Sugar-sweetened beverages	Low	464	51.8
	High	431	48.2
Late-night eating	Low	772	86.3
	High	123	13.7
Regular dining	Yes	752	84.0
	No	143	16.0
Physical activity	Low	326	36.4
	High	569	63.6
Pressure perception	Yes	206	23.0
	No	689	77.0
Sleep duration	Above 6 h/d	830	92.7
	≤ 6 h/d	65	7.3
Screen time	Above 2 h/d	798	89.2
	≥ 2 h/d	97	10.8

### Identification of Behavior Profiles

**Figure 1:**
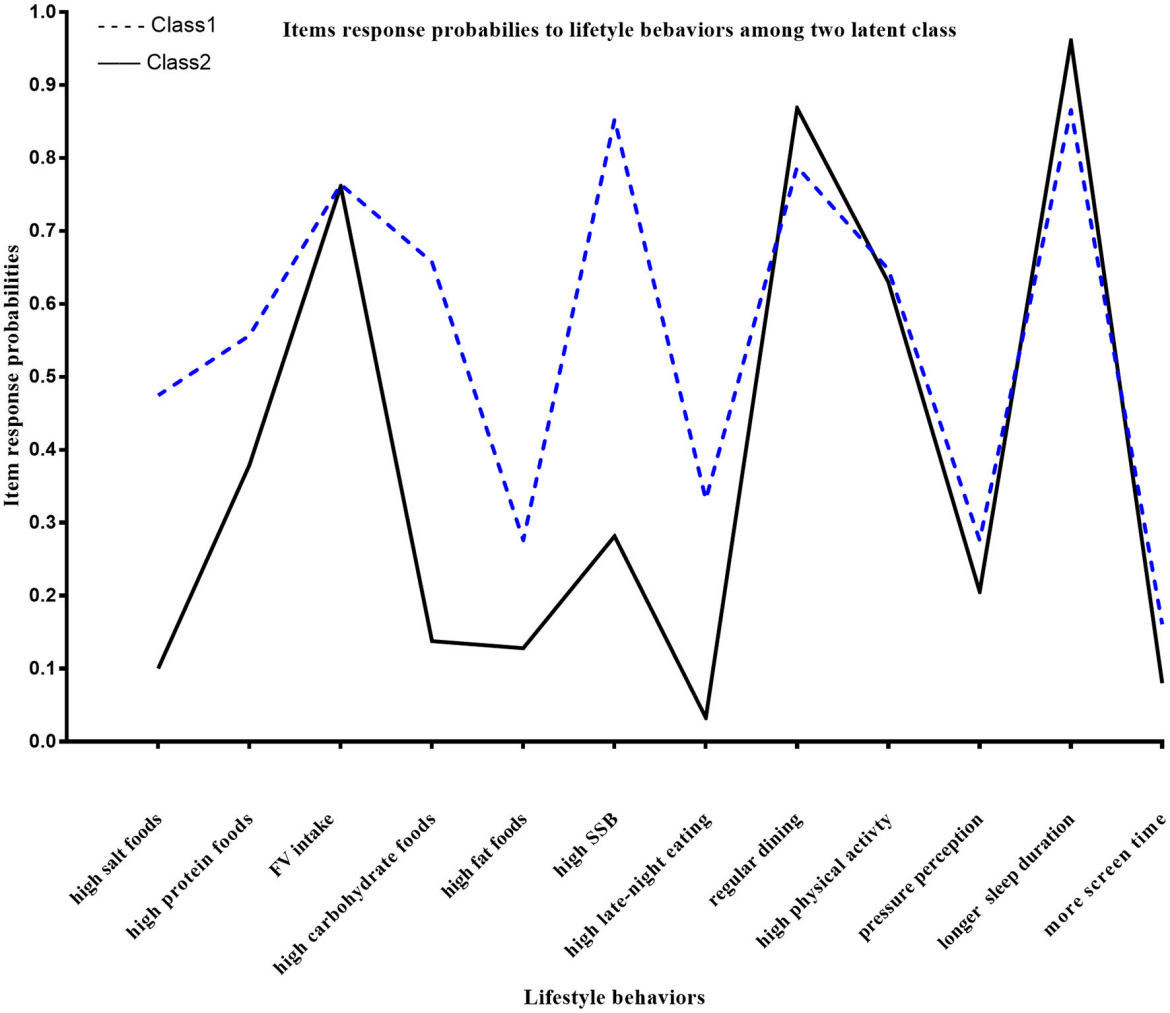
Response probabilities to lifestyle behaviors in the two classes: Class 1: sub-healthy lifestyle group; Class 2: healthy lifestyle group: high-salt foods: consumption of salt-rich foods at least three times per week; high-protein foods: protein-rich foods at least three times per week; FV intake: fruit and vegetables at least three times per day; high-carbohydrate foods: carbohydrate rich foods at least three times per week; high-fat foods: fat-rich foods at least three times per week; high SSB: sugar-sweetened beverages at least three times per week; high late-night eating: snacks at night for at least 3 days per week; regular dining: three meals per day at a relatively fixed time; high physical activity: PA that lasted at least 30 min for at least 3 days in the past 7 days; pressure perception: felt stressed up from studies or daily life; longer sleep duration: ≥ 6 h of usual time for sleeping at night; more screen time: ≥ 2 h of total time spent per day using electronic devices.

Fit indices for the model based on the 12 lifestyle behaviors are shown in Table 2. The model with four latent classes had the lowest AIC (10776.5), adjusted BIC (10859.2), and highest entropy (entropy = 0.643). A two-class model had the lowest BIC (10,972.14). The BLRT results indicated that there was no significant difference between the three and two-class models (*p* = 0.213). Compared with other models, a two-class model had overall superior metrics and also enabled better interpretability of classes, thus providing the best fit. The two classes identified in this study were “sub-healthy lifestyle” (class 1) and “healthy lifestyle” (class 2). Class 2 had an overall healthier behavior profile compared to class 1. Class 1 involved 34.2% of the population and was characterized by a high proportion of adolescents reporting a high-salt, -carbohydrate, and -fat diet (47.5, 65.7, and 27.6%, respectively). The corresponding rates in Class 2 were 10, 13.8, and 12.8%, respectively. Class 1 also included a high proportion of participants who consumed SSB (85.3%) and snacks at night (33.2%). Class 2 involved 65.8% of the population and was characterized by a large number of participants with healthy behaviors (Supplementary Table S3). This was indicated by a higher proportion of participants reporting regular dining (86.9%) and refraining from high-salt, -carbohydrate, and fat-rich foods (90.0, 86.2, and 87.2%, respectively). This group also had a longer sleeping duration (96.1%), less screen time (8.0%), and less pressure-perception (20.5%) (Figure 1).

**Table 2:** Test of goodness of fit for latent class model.

**Model**	**L2, *P***	**AIC**	**BIC**	**aBIC**	**BLRT, *P***	**Entropy**
2-cluster	−5401.11, *p =* 1.0	10,852.2	**10,972.14**	10,892.8	**−5,552.95**, ***p =*** **0.000**	0.590
3-cluster	−5362.50, *p =* 1.0	10,801.0	10,983.28	10,862.6	−5,401.11, *p =* 0.213	0.589
4-cluster	−5337.26, *p =* 1.0	10,776.5	11,021.15	10,859.2	−5,362.50, *p =* 0.476	0.643

*AIC, Akaike's information criterion; BIC, Bayesian information criteria; aBIC, adjusted Bayesian information criteria; BLRT, bootstrap likelihood ratio tests*.

*The bold values represent that a two-class model had the lowest BIC (10,972.14) and the BLRT results indicated that there was significant difference between the one and two-class models (p = 0.000)*.

### Association Between Class for the Final Two-Class Solution and Obesity-Related Cardiometabolic Outcomes

The characteristics of the two classes are summarized in Table 3. In total, 43.0% of the adolescents aged 15–16 years had a “healthy lifestyle,” whereas 63.7% of the adolescents aged 16–17 years had a “sub-healthy lifestyle”. A healthy lifestyle was more common in females than in males (56.2 vs. 43.8%), whereas a sub-healthy lifestyle was more common in males (54.6 vs. 45.4%), (all P = 0.002). Compared with the Class 2, the Class 1 group showed no significantly difference in the proportion of normal-weight participants (78.8 vs. 81.7%, P =0.475). There was no significant difference in obesity-related cardiometabolic risk factors between the two classes, except for WC (70.5 vs. 69.1 cm, P = 0.044). Increased risk of obesity and hypertension was not significant across different lifestyle patterns in the logistic regression models (odds ratio (OR) 1.11;95%CI 0.78–1.57, 1.09; 95%CI 0.73–1.52, and 0.96; 95%CI 0.64–1.43, respectively), as shown in Table 4.

**Table 3:** Demographic and cardiovascular markers for each of the two clusters.

**Variables**	**Class 1**	**Class 2**	**χ2/ z**	**P**
Age group, years				
14–15 years	4 (1.3%)	4 (0.7%)	9.023	**0.029**
15–16 years	101 (33.0%)	253 (43.0%)		
16–17 years	195 (63.7%)	320 (54.3%)		
17–18 years	6 (2.0%)	12 (2.0%)		
Sex				
Male	167 (54.6%)	258 (43.8%)	9.371	**0.002**
Female	139 (45.4%)	331 (56.2%)		
Ethnic				
Han	130 (42.5%)	130 (42.5%)	0.143	0.706
Others	130 (42.5%)	130 (42.5%)		
Weight status				
Obesity	17 (5.6%)	33 (5.6%)	1.489	0.475
Overweight	48 (15.7%)	75 (12.7%)		
Normal	241 (78.8%)	481 (81.7%)		
Central obesity				
Yes	64 (20.9%)	131 (22.2%)	0.208	0.648
No	242 (79.1%)	458 (77.8%)		
Hypertension				
Yes	44 (14.4%)	82 (13.9%)	0.035	0.852
No	262 (85.6%)	507 (86.1%)		
Family history				
Yes	120 (39.2%)	234 (39.7%)	0.022	0.882
No	186 (60.8%)	355 (60.3%)		
WC, cm ^#^	70.5 (66.2,76.5)	69.1 (65.2,75.8)	82,725.5	**0.044**
BMI, kg/m^2^ ^#^	20.5 (18.8,23.0)	20.6 (19.0,22.4)	89,155.0	0.793
SBP, mmHg ^#^	112 (105,120)	111 (103,118)	83,632.0	0.077
DBP, mmHg ^#^	69 (65,75)	70 (65,76)	85,513.0	0.209
TC, mmol/L (n = 592) ^#^	4.14 (3.74,4.62)	4.11 (3.67,4.53)	−0.885	0.376
TG, mmol/L (n = 592) ^#^	0.99 (0.80,1.47)	0.97 (0.75,1.41)	−1.196	0.232
HDL-C, mmol/L (n = 592) ^#^	1.23 (1.05,1.42)	1.26 (1.06,1.48)	−0.507	0.612
LDH-C, mmol/L (n = 592) ^#^	2.30 (2.02,2.87)	2.30 (1.96,2.70)	−0.76	0.447

# *, data was presented as median (interquartile ranges) for variables with skewed distribution*.

*Bold number indicates p < 0.05. Sample sizes differ because 592 subjects who consented to undergo blood tests were included for the lipids analysis*.

*WC, waist circumference; BMI, body mass index; SBP, Systolic blood pressure; DBP, diastolic blood pressure; TC, total cholesterol; TG, triglyceride; HDL-C, high-density lipoprotein cholesterol; LDL-C, low-density lipoprotein cholesterol*.

**Table 4:** The association of the latent class with obesity and hypertension.

	**OR**	***P***	OR^*^	P^*^
Obesity				
Class 1	1.20 (0.85, 1.70)	0.297	1.11 (0.78, 1.57)	0.569
Class 2	1.0		1.0	
Central obesity				
Class 1	1.08 (0.77, 1.52)	0.649	1.09 (0.73, 1.52)	0.638
Class 2	1.0		1.0	
Hypertension				
Class 1	1.04 (0.70, 1.54)	0.852	0.96 (0.64, 1.43)	0.833
Class 2	1.0		1.0	

* *adjusted for sex and age*.

*OR, odds ratios, Class 1, sub-healthy lifestyle group; Class 2, healthy lifestyle group*.

### Analysis of the Characteristics That Distinguished Each Class

The analysis showed that most lifestyle behaviors, including dietary intake, sleep duration, and pressure perception, differed substantially between the two classes. However, there was no significant between-class difference in PA and fruit and vegetables (FV)-rich diet (Supplementary Table S4). A higher proportion of individuals consumed protein-rich food in the sub-healthy behavior group than in the healthy group (55.9 vs. 38.0%, *P* < 0.001).

## Discussion

Although lifestyle is an established risk factor of CVD, previous studies have focused on the association of a specific lifestyle behavior instead of the overall lifestyle. This study identified two lifestyle patterns: a healthy behavior pattern and a sub-healthy behavior pattern. Importantly, there were significant differences in most lifestyle behaviors, including dietary intake, sleep duration, screen time, and stress perception between the two classes. However, there were no significant between-class differences in PA and FV intake. Adolescents who demonstrated healthy behavior patterns consumed a low amount of food rich in salt, fats, and carbohydrates, and slept longer, ate fewer late-night snacks, and had a shorter screen time. Additionally, they consumed more regular meals and were subjected to peer pressure to a lower extent. However, different lifestyle clusters were not significantly associated with obesity, blood pressure, and lipid profiles in the present study.

A previous study on the genetic and environmental factors of hypertension in high school students found a significant association between BP values and urinary sodium excretion (30), in contrast to the findings among adults (31). The results of this study can also be explained by the same potential mechanism: the compensatory capacity of the body in early life masks these invisible cardiovascular complications caused by unfavorable lifestyle factors, but these compensatory mechanisms gradually lose efficiency in later life. Another explanation might be that the differential gene expression between the environment and the susceptible gene host background determines the phenotype during this period (32). Surprisingly, our findings appear to provide novel information about the drivers of abnormal cardiometabolic outcomes in adolescence rather than confirming some expected relationships. The two latent classes showed no significant difference in PA and FV intake, and this may account for the lack of significant difference in obesity-related cardiovascular abnormalities. A cross-sectional and longitudinal cohort study (33) by Richard et al. showed that PA, moderate and vigorous PA (MVPA), and sedentary time (ST) were significant contributors to variations in body fat, whereas total energy, sugar, and fat intake were not.

Another community-based longitudinal study of Australian children aged 4 to 15 years (Parent Education and Support (PEAS) Kids Growth study) suggested that decade-long dietary trajectories in healthy children do not influence macro- or microvascular structure or stiffness in mid-adolescence (17). The study also found that the quality of the children's diet changed between 6.5 and 10 years of age, with marked early-life differences in diet quality becoming slightly less in adolescence. Collectively, these studies may suggest that diet factors in adolescents are less essential than previously thought. Meanwhile, PA is known to be strongly associated with obesity in the traditional independent variable analysis in adults and children (34, 35), it remains unclear whether the alike effect of different lifestyle pattern on cardiometabolic outcomes is due to the model's failure to distinguish PA variable. Or we can speculate that PA may be a key trigger of cardiovascular change during this period. In addition, there was no significant difference in FV intake between the two latent classes. However, this could be because we could not collect on information on the exact quantity and variety of foods consumed, leading to misclassification of the adolescents in the LCA. FV intake has been proposed to have favorable effects on obesity and cardiometabolic markers in adults. However, it remains unclear whether the influence of FV intake on adiposity among adolescents is independent of decreased caloric intake and increased PA (36). Notably, we found a higher proportion of individuals who consumed protein-rich food in the sub-healthy behavior group than in the healthy group (55.9 vs. 38.0%), which the only favorable factor was common in the sub-healthy behavior group. Although a recent meta-analysis found that protein intake is correlated with the risk of all-cause mortality, cardiovascular disease, and cancer (37), we speculated this single factor hardly offsets the effect of the sum of all other factors.

Research in the past few years has shifted toward modifying a combination of multiple lifestyle components representing the overall lifestyle behaviors. A composite healthy lifestyle score was constructed for adults based on the American Heart Association cardiovascular health recommendations in 2010 (38), but this has been rarely applied in studies on children and adolescents. A recent cross-sectional and prospective study of 1,480 children from the Infancia y Medio Ambiente [Environment and Childhood] birth cohort constructed a child lifestyle score by summing five behaviors (PA, sleep time, television time, plant-based foods, and intake of ultra-processed foods) at the age of 4 years. The results showed that the lifestyle score was not associated with obesity and cardiometabolic risk at baseline, however, it was negatively associated with BMI and WC Z-scores at the age of 7 years (39). Several studies also used cluster analysis to comprehensively explore adolescent lifestyle behaviors with variables such as PA, sedentary behaviors, screen time, and sleep duration included in the analysis (40–42). Notable, a Spanish study of 207 Spanish children aged between 9–12 years and 208 adolescents aged between 13–17 years identified two different lifestyle groups using hierarchical clustering analysis (40). The unhealthier lifestyle pattern comprised low PA and a poor dietary pattern, and healthier lifestyle pattern included high PA, low sedentary behavior, longer sleep duration, and a healthier dietary pattern. However, increased risk of being overweight was not significant across lifestyle patterns in children and adolescents (Prevalence ratio 1.07;95%CI 0.55–2.04 and 2.00;95%CI 0.82–4.86, respectively).

Rosaura et al. also established three differential clusters based on physical and sedentary activities and lifestyle behaviors. Cluster 1 children (with the mostly sedentary state) exhibited higher insulin, Homeostatic Model Assessment for Insulin Resistance, and triglyceride levels (42). Despite clear differences in approaches and interpretations, these data might offer more insight into the overall lifestyle behaviors of children. LCA is an exploratory statistical modeling method, and there is no definitive test to facilitate the identification of the “true” number of latent classes, with different categories based on various observed variables. Several cross-sectional and longitudinal studies have demonstrated that many lifestyle behaviors co-occur, and distinct profiles that predict obesity have been identified using lifestyle behavior variables in children, adolescents, and young adults (19–24). To the best of our knowledge, the latent subtypes of lifestyle behaviors in adolescent obesity were first investigated in 2010 (19). The results generally suggest that LCA is an effective and valid method in categorizing individuals with similar characteristics.

Lifestyle behaviors vary according to socio-economic and ethnic backgrounds, but data for Chinese adolescents are limited. Our school-based population reported an extremely low (or hardly any) prevalence of high-risk behaviors (smoking, drinking, abortion, and drug abuse) and was relatively homogeneous. Therefore, we could select common behaviors as study variables. Recently, a Chinese study exploring the relationship between weight misperception and lifestyle behaviors among 10,708 Chinese children and adolescents derived three latent dietary and activity patterns, namely, obesogenic pattern, malnourished pattern, and healthy pattern (24). This may be because PA was categorized based on the current recommendations of <60 min/day and ≥60 min/day, whereas we used a cutoff of 30 min per day at least 3 days per week. Therefore, the prevalence of PA was higher in our study (63.6%). This difference can be attributed to the creation of categories based on the frequency at which behaviors were performed and which foods were consumed, leading to a different classification of adolescents in the LCA. This study was designed to explore the number of latent classes fitted and the association between clusters and cardiovascular markers through the latent class model using different observed variables. Regretfully, increased risk of cardiometabolic abnormality was not significant across different lifestyle patterns. Due to the complexity effect of lifestyle clusters on cardiometabolic risks, prospective and well-designed studies are needed in the future.

A national survey of 131,859 students aged between 7 and 19 years from 986 public schools in China demonstrated that 34.1% of them met the MVPA definition (≥60 min of MVPA per day) and 65.4% adhered to the screen time guideline of 2 h daily (43). Along with a fourfold increase in overweight and obesity rates in the youth, improving the health of children and adolescents has become a national priority. Lack of PA is regarded as a stumbling block. Unfortunately, research to date suggests that diet and exercise prescription has a generally small to moderate effect on preventing obesity and cardiometabolic risk (44, 45). This might be improved if individualized prevention strategies are targeted toward subgroups at the greatest risk. Adolescents have a high nutritional requirement (46), but a highly restrictive diet may have adverse effects. We recommend that the strategies should be targeted toward increasing PA, rather than dietary restrictions in adolescents.

The major strength of this study was that there were no predefined hypotheses made, therefore, a *post hoc* analysis was used to statistically examine the effect of lifestyle behaviors, and all the results are exploratory. Additionally, to the best of our knowledge, this is the first study to use LCA to examine the association of lifestyle behaviors with blood pressure and lipid profile. Furthermore, the large sample size and relatively homogenous nature of our population improved the statistical significance of the results. However, our study also has some limitations. First, the latent classes were not as well discriminated as we would like, as evidenced by the moderate entropy and partially overlapping risk profiles. Second, instead of a valid and reliable measurement, we used self-reported lifestyle variables and these may be affected by the response and recall bias. The dietary data collected using a semi-quantitative FFQ could also have been lacking in precision. Dichotomous variables could have been misclassified into any possible subcategory, although it enabled an easier interpretation of the model. Pressure perception was assessed through a subjective question in the self-designed questionnaire. Currently, there are some scales applicable to children that can assess emotional and psychological states, such as Self-esteem scales (SES) and Depression scales. However, they were not used in this study. This is also a limitation of this exploratory study. Third, the findings may have limited generalizability because of the single-center design. Lastly, a causal relationship cannot be assumed because of the cross-sectional study design. Prospective studies are needed to validate our findings.

In conclusion, primary prevention based on lifestyle modification should target patterns of behaviors at high risk in adolescents, as this might be more effective in preventing and treating future NCCDs. Due to the complex effect of lifestyle clusters on cardiometabolic risks, well-designed and prospective studies in adolescents are needed in the future.

## Data Availability Statement

The original contributions presented in the study are included in the article/Supplementary Material, further inquiries can be directed to the corresponding author.

## Ethics Statement

The studies involving human participants were reviewed and approved by the Clinical Ethical Committee of The First Affiliated Hospital of Guangxi Medical University. Written informed consent to participate in this study was provided by the participants' legal guardian/next of kin.

## Author Contributions

WZ and YP contributed to research conception, design, and drafted the article. LM and JL contributed to data acquisition. WZ and LM contributed to data analysis and interpretation. DS contributed to sensitivity analysis and interpretation of the revised manuscript. DS, YP, CC, BY, and SQ revised the article. All authors were involved in writing the article and had final approval of the submitted and published versions.

## Conflict of Interest

The authors declare that the research was conducted in the absence of any commercial or financial relationships that could be construed as a potential conflict of interest.

## Publisher's Note

All claims expressed in this article are solely those of the authors and do not necessarily represent those of their affiliated organizations, or those of the publisher, the editors and the reviewers. Any product that may be evaluated in this article, or claim that may be made by its manufacturer, is not guaranteed or endorsed by the publisher.

## References

[1] Chung ST Onuzuruike AU Magge SN. Cardiometabolic risk in obese children. Ann N Y Acad Sci. (2018) 1411:166–83. 10.1111/nyas.13602 29377201 PMC5931397

[2] Wuhl E. Hypertension in childhood obesity. Acta paediatrica (Oslo, Norway: 1992). (2019) 108(1):37–43. 10.1111/apa.14551 30144170

[3] Yang L Magnussen CG Yang L Bovet P Xi B. Elevated Blood Pressure in Childhood or Adolescence and Cardiovascular Outcomes in Adulthood: A Systematic Review. Hypertension (Dallas, Tex: 1979). (2020) 75:948–55. 10.1161/hypertensionaha.119.14168 32114851

[4] Elkins C Fruh S Jones L Bydalek K. Clinical Practice Recommendations for Pediatric Dyslipidemia. J Pediatr Health Care. (2019) 33:494–504. 10.1016/j.pedhc.2019.02.009 31227123

[5] Allen NB Krefman AE Labarthe D Greenland P Juonala M Kähönen M. Cardiovascular health trajectories from childhood through middle age and their association with subclinical atherosclerosis. JAMA Cardiol. (2020) 5:557–66. 10.1001/jamacardio.2020.0140 32159727 PMC7066520

[6] Afshin A Forouzanfar MH Reitsma MB Sur P Estep K Lee A. Health effects of overweight and obesity in 195 countries over 25 years. N Engl J Med. (2017) 377:13–27. 10.1056/NEJMoa1614362 28604169 PMC5477817

[7] Weihe P Weihrauch-Blüher S. Metabolic syndrome in children and adolescents: diagnostic criteria, therapeutic options and perspectives. Curr Obes Rep. (2019) 8:472–9. 10.1007/s13679-019-00357-x 31691175

[8] DeBoer MD. Assessing and managing the metabolic syndrome in children and adolescents. Nutrients. (2019). 11:1788. 10.3390/nu11081788 31382417 PMC6723651

[9] Yang L Kelishadi R Hong YM Khadilkar A Nawarycz T Krzywińska-Wiewiorowska M. Impact of the 2017. American academy of pediatrics guideline on hypertension prevalence compared with the fourth report in an international cohort. Hypertension (Dallas, Tex: 1979). (2019). 74:1343–8. 10.1161/hypertensionaha.119.13807 31630571

[10] Wang L Song L Liu B Zhang L Wu M Cao Z. Trends and Status of the Prevalence of Elevated Blood Pressure in Children and Adolescents in China: a Systematic Review and Meta-analysis. Curr Hypertens Rep. (2019) 21:88–88. 10.1007/s11906-019-0992-1 31599364

[11] Imam MU Ismail M. The impact of traditional food and lifestyle behavior on epigenetic burden of chronic disease. Global Challenges (Hoboken, NJ). (2017) 1:1700043. 10.1002/gch2.201700043 31565292 PMC6607231

[12] Abrignani MG Lucà F Favilli S Benvenuto M Rao CM Di Fusco SA. Lifestyles and cardiovascular prevention in childhood and adolescence. Pediatr Cardiol. (2019) 40:1113–25. 10.1007/s00246-019-02152-w 31342115

[13] Leech RM McNaughton SA Timperio A. The clustering of diet, physical activity and sedentary behavior in children and adolescents: a review. Int J Behav Nutr Phys Act. (2014) 11:4. 10.1186/1479-5868-11-4 24450617 PMC3904164

[14] Parker KE Salmon J Costigan SA Villanueva K Brown HL Timperio A. Activity-related behavior typologies in youth: a systematic review. Int J Behav Nutr Phys Act. (2019) 16:44. 10.1186/s12966-019-0804-7 31097036 PMC6524235

[15] Navarro P Shivappa N Hébert JR Mehegan J Murrin CM Kelleher CC. Predictors of the dietary inflammatory index in children and associations with childhood weight status: A longitudinal analysis in the Lifeways Cross-Generation Cohort Study. Clin Nutr. (2020) 39:2169–79. 10.1016/j.clnu.2019.09.004 31606243

[16] Kupek E Lobo AS Leal DB Bellisle F de Assis MA. Dietary patterns associated with overweight and obesity among Brazilian schoolchildren: an approach based on the time-of-day of eating events. Br J Nutr. (2016) 116:1954–65. 10.1017/s0007114516004128 27976603

[17] Kerr JA Gillespie AN Gasser CE Mensah FK Burgner D Wake M. Childhood dietary trajectories and adolescent cardiovascular phenotypes: Australian community-based longitudinal study. Public Health Nutr. (2018) 21:2642–53. 10.1017/s1368980018001398 29947308 PMC10260830

[18] Lanza ST Rhoades BL. Latent class analysis: an alternative perspective on subgroup analysis in prevention and treatment. Prev Sci. (2013) 14:157–68. 10.1007/s11121-011-0201-1 21318625 PMC3173585

[19] Huh J Riggs NR Spruijt-Metz D Chou CP Huang Z Pentz M. Identifying patterns of eating and physical activity in children: a latent class analysis of obesity risk. Obesity (Silver Spring, Md). (2011) 19:652–8. 10.1038/oby.2010.228 20930718 PMC5310931

[20] Laxer RE Brownson RC Dubin JA Cooke M Chaurasia A Leatherdale ST. Clustering of risk-related modifiable behaviours and their association with overweight and obesity among a large sample of youth in the COMPASS study. BMC Public Health. (2017) 17:102. 10.1186/s12889-017-4034-0 28109270 PMC5251243

[21] Magee CA Caputi P Iverson DC. Patterns of health behaviours predict obesity in Australian children. J Paediatr Child Health. (2013) 49:291–6. 10.1111/jpc.12163 23574555

[22] Hendryx M Chojenta C Byles JE. Obesity Risk Among Young Australian Women: A Prospective Latent Class Analysis. Obesity (Silver Spring, Md). (2020) 28:154–60. 10.1002/oby.22646 31755240

[23] Miranda VPN Dos Santos Amorim PR Bastos RR Souza VGB de Faria ER do Carmo Castro Franceschini S. Evaluation of lifestyle of female adolescents through latent class analysis approach. BMC Public Health. (2019) 19:184. 10.1186/s12889-019-6488-8 30760240 PMC6373094

[24] Qin TT Xiong HG Yan MM Sun T Qian L Yin P. Body weight misperception and weight disorders among Chinese children and adolescents: a latent class analysis. Curr Med Sci. (2019) 39:852–62. 10.1007/s11596-019-2116-1 31612407

[25] Das JK Salam RA Thornburg KL Prentice AM Campisi S Lassi ZS Nutrition in adolescents: physiology, metabolism, and nutritional needs. Ann N Y Acad Sci. (2017) 1393:21–33. 10.1111/nyas.13330 28436102

[26] Chen MY Wang EK Jeng YJ. Adequate sleep among adolescents is positively associated with health status and health-related behaviors. BMC Public Health. (2006) 6:59. 16524482 10.1186/1471-2458-6-59 PMC1447528

[27] Group of China Obesity Task Force. [Body mass index reference norm for screening overweight and obesity in Chinese children and adolescents]. Zhonghua Liu Xing Bing Xue Za Zhi. (2004) 25:97–102. 15132858

[28] Ma GS Ji CY Ma J Mi J Sung R Xiong F. [Waist circumference reference values for screening cardiovascular risk factors in Chinese children and adolescents aged 7 - 18 years]. Zhonghua Liu Xing Bing Xue Za Zhi. (2010) 31:609–15. 21163088

[29] Dong Y Ma J Song Y Dong B Wang Z Yang Z. National blood pressure reference for Chinese han children and adolescents aged 7 to 17 years. Hypertension (Dallas, Tex: 1979). (2017) 70:897–906. 10.1161/hypertensionaha.117.09983 28923902 PMC5722224

[30] Bigazzi R Zagato L Lanzani C Fontana S Messaggio E Delli Carpini S. Hypertension in high school students: genetic and environmental factors: The HYGEF study. Hypertension (Dallas, Tex: 1979) 75:71–8. 10.1161/hypertensionaha.119.13818 31760884

[31] Sacks FM Svetkey LP Vollmer WM Appel LJ Bray GA Harsha D. Effects on blood pressure of reduced dietary sodium and the Dietary Approaches to Stop Hypertension (DASH) diet. N Engl J Med. (2001) 344:3–10. 10.1056/nejm200101043440101 11136953

[32] Plaza-Florido A Altmäe S Esteban FJ Cadenas-Sanchez C Aguilera CM Einarsdottir E. Distinct whole-blood transcriptome profile of children with metabolic healthy overweight/obesity compared to metabolic unhealthy overweight/obesity. Pediatr Res. (2020) 89:1687–94. 10.1038/s41390-020-01276-7 33230195

[33] Telford RD Telford RM Martin MK Welvaert M. Drivers of adolescent adiposity: Evidence from the Australian LOOK study. J Sci Med Sport. (2019) 22:1330–4. 10.1016/j.jsams.2019.07.013 31445949

[34] Poorolajal J Sahraei F Mohamdadi Y Doosti-Irani A Moradi L. Behavioral factors influencing childhood obesity: a systematic review and meta-analysis. Obes Res Clin Pract. (2020) 14:109–18. 10.1016/j.orcp.2020.03.002 32199860

[35] Huai P Xun H Reilly KH Wang Y Ma W Xi Xi B: Physical activity and risk of hypertension: a meta-analysis of prospective cohort studies. Hypertension (Dallas, Tex: 1979). (2013) 62:1021–6. 10.1161/HYPERTENSIONAHA.113.01965 24082054

[36] Ledoux TA Hingle MD Baranowski T. Relationship of fruit and vegetable intake with adiposity: a systematic review. Obes Rev. (2011) 12:e143–150. 10.1111/j.1467-789X.2010.00786.x 20633234

[37] Naghshi S Sadeghi O Willett WC Esmaillzadeh A. Dietary intake of total, animal, and plant proteins and risk of all cause, cardiovascular, and cancer mortality: systematic review and dose-response meta-analysis of prospective cohort studies. BMJ (Clinical Research ed). (2020) 370:m2412. 10.1136/bmj.m2412 32699048 PMC7374797

[38] Lloyd-Jones DM Hong Y Labarthe D Mozaffarian D Appel LJ Van Horn L Defining and setting national goals for cardiovascular health promotion and disease reduction: the American Heart Association's strategic Impact Goal through 2020 and beyond. Circulation. (2010) 121:586–613. 10.1161/circulationaha.109.192703 20089546

[39] Bawaked RA Fernández-Barrés S Navarrete-Muñoz EM González-Palacios S Guxens M Irizar A Impact of lifestyle behaviors in early childhood on obesity and cardiometabolic risk in children: Results from the Spanish INMA birth cohort study. Pediatr Obes. (2020) 15:e12590. 10.1111/ijpo.12590 31793235

[40] Pérez-Rodrigo C Gil Á González-Gross M Ortega RM Serra-Majem L Varela-Moreiras G. Clustering of dietary patterns, lifestyles, and overweight among Spanish children and adolescents in the ANIBES study. Nutrients. (2015) 8:11. 10.3390/nu8010011 26729155 PMC4728625

[41] Dumuid D Olds T Martín-Fernández JA Lewis LK Cassidy L Maher C. Academic performance and lifestyle behaviors in Australian school children: a cluster analysis. Health Educ Behav. (2017) 44:918–27. 10.1177/1090198117699508 28436241

[42] Leis R Jurado-Castro JM Llorente-Cantarero FJ Anguita-Ruiz A Iris-Rupérez A Bedoya-Carpente JJ. Cluster analysis of physical activity patterns, and relationship with sedentary behavior and healthy lifestyles in prepubertal children: genobox cohort. Nutrients. (2020) 12:1288. 10.3390/nu12051288 32370020 PMC7282254

[43] Chen P Wang D Shen H Yu L Gao Q Mao L. Physical activity and health in Chinese children and adolescents: expert consensus statement (2020). Br J Sports Med. (2020) 54:1321–31. 10.1136/bjsports-2020-102261 32471813 PMC7606574

[44] Ho M Garnett SP Baur L Burrows T Stewart L Neve M. Effectiveness of lifestyle interventions in child obesity: systematic review with meta-analysis. Pediatrics. (2012) 130:e1647–1671. 10.1542/peds.2012-1176 23166346

[45] Oosterhoff M Joore M Ferreira I. The effects of school-based lifestyle interventions on body mass index and blood pressure: a multivariate multilevel meta-analysis of randomized controlled trials. Obes Rev. (2016) 17:1131–53. 10.1111/obr.12446 27432468

[46] Christian P Smith ER. Adolescent undernutrition: global burden, physiology, and nutritional risks. Ann Nutr Metab. (2018) 72:316–28. 10.1159/000488865 29730657

