# Supplementary material for: Lifestyle Clusters and Cardiometabolic Risks in Adolescents: A Chinese School-Based Study Using a Latent Class Analysis Approach
Source: Front Pediatr. 2021 Dec 16;9:728841. doi: 10.3389/fped.2021.728841 (PMC8716941; doi:10.3389/fped.2021.728841)
Supplement: Supplementary file 1 [file Data_Sheet_1.doc]

Table S1 The code used for LCA in Mplus8.3 software

| High-salt foods | （1=Low，2=High） |
| --- | --- |
| High-protein foods | （1=Low，2=High） |
| FV intake | （1=Low，2=High） |
| High-carbohydrate foods | （1=Low，2=High） |
| High-fat foods | （1=Low，2=High） |
| SSB consumption | （1=Low，2=High） |
| Late-night eating | （1=Low，2=High） |
| Regular dining | （1=Yes，2=No） |
| Physical activity | （1=Low，2=High） |
| Pressure perception | （1=Yes，2=No） |
| Sleep duration | （1＞6h/d，2≤6h/d） |
| Screen time | （1＜2h/d，2≥2h/d） |

Table S2 The basic characteristics between participants and non-participants

|  |  | **The excluded (n=396)** | **The include (n=895)** | ***P*** |
| --- | --- | --- | --- | --- |
| Sex | Female | 208(52.5%) | 425(47.5%) | 0.095 |
|  | Male | 188(47.5%) | 470(52.5%) |  |
| Age,years |  | 16.0(15.7，16.3) | 16.0(15.8,16.3) | 0.750 |
| BMI,kg/m2 |  | 20.46(18.65,23.53) | 20.55(18.88,22.65) | 0.865 |
| SBP,mmHg |  | 112(104,120) | 111(104,119) | 0.674 |
| DBP,mmHg |  | 69(65,75) | 75(65,75) | 0.310 |

Table S3 Conditional probabilities and latent class probabilities for healthy behaviors

| **Observed variable** | **Category** | **Latent variable** | |
| --- | --- | --- | --- |
|  |  | class1 | class2 |
| High-salt foods | Low | 0.5254 | 0.8998 |
|  | High | 0.4746 | 0.1002 |
| High-protein foods | Low | 0.4435 | 0.6207 |
|  | High | 0.5565 | 0.3793 |
| FV intake | Low | 0.2363 | 0.2389 |
|  | High | 0.7637 | 0.7611 |
| High-carbohydrate foods | Low | 0.3428 | 0.8623 |
|  | High | 0.6572 | 0.1377 |
| High-fat foods | Low | 0.7241 | 0.8719 |
|  | High | 0.2759 | 0.1281 |
| SSB consumption | Low | 0.1471 | 0.7185 |
|  | High | 0.8529 | 0.2815 |
| Late-night eating | Low | 0.6681 | 0.9673 |
|  | High | 0.3319 | 0.0327 |
| Regular dining | Yes | 0.7877 | 0.8685 |
|  | No | 0.2123 | 0.1315 |
| Physical activity | Low | 0.3529 | 0.3703 |
|  | High | 0.6471 | 0.6297 |
| Pressure perception | Yes | 0.2760 | 0.2055 |
|  | No | 0.7240 | 0.7945 |
| Sleep duration | ＞6 hours/d | 0.8657 | 0.9606 |
|  | ≤6 hours/d | 0.1343 | 0.0394 |
| Screen time | ＜2h/d | 0.8392 | 0.9199 |
|  | ≥2h/d | 0.1608 | 0.0801 |
| Latent class probabilities |  | 0.3419 | 0.6581 |

Table S4 Behavioral characteristics of two latent classes in the study

| **Variables** |  | **class1(%)** | **class2(%)** | ***χ2*** | ***P*** |
| --- | --- | --- | --- | --- | --- |
| High-salt foods | Low | 146(47.7) | 542(92.0) | 222.364 | **＜0.001** |
|  | High | 160(52.3) | 47(8.0) |  |  |
| High-protein foods | Low | 135(44.1) | 365(62.0) | 26.029 | **＜0.001** |
|  | High | 171(55.9) | 224(38.0) |  |  |
| FV intake | Low | 75(24.5) | 138(23.4) | 0.130 | 0.719 |
|  | High | 231(75.5) | 451(76.6) |  |  |
| High-carbohydrate foods | Low | 86(28.1) | 523(88.8) | 341.123 | **＜0.001** |
|  | High | 220(71.9) | 66(11.2) |  |  |
| High-fat foods | Low | 227(74.2) | 507(86.1) | 19.314 | **＜0.001** |
|  | High | 79(25.8) | 82(13.9) |  |  |
| SSB | Low | 25(8.2) | 439(74.5) | 355.237 | **＜0.001** |
|  | High | 281(91.8) | 150(25.5) |  |  |
| Late-night eating | Low | 198(64.7) | 574(97.5) | 182.176 | **＜0.001** |
|  | High | 108(35.3) | 15(2.5) |  |  |
| Regular dining | No | 65(21.2) | 78(13.2) | 9.060 | **0.002** |
|  | Yes | 241(78.8) | 511(86.8) |  |  |
| Physical activity | Low | 112(36.6) | 214(36.3) | 0.006 | 0.937 |
|  | High | 194(63.4) | 375(63.7) |  |  |
| Pressure perception | Yes | 86(28.1) | 120(20.4) | 6.793 | **0.009** |
|  | No | 220(71.9) | 469(79.6) |  |  |
| Sleep duration | ＞6 hours/d | 266(86.9) | 564(95.8) | 23.298 | **＜0.001** |
|  | ≤6 hours/d | 40(13.1) | 25(4.2) |  |  |
| Screen time | ＜2h/d | 256(83.7) | 542(92.0) | 14.565 | **＜0.001** |
|  | ≥2h/d | 50(16.3) | 47(8.0) |  |  |
